# HEARING LOSS AND ITS CONSEQUENCES FOR SPOUSAL MENTAL HEALTH: EVIDENCE FROM THE HEALTH AND RETIREMENT STUDY

**DOI:** 10.1093/geroni/igac059.1692

**Published:** 2022-12-20

**Authors:** Jessica West, Sherri Smith, Matthew Dupre

**Affiliations:** Duke University, Durham, North Carolina, United States; Duke University, Durham, North Carolina, United States; Duke University, Durham, North Carolina, United States

## Abstract

Hearing loss (HL) is an increasingly prevalent chronic stressor among older adults and is associated with numerous adverse health outcomes. The life course perspective and stress process framework highlight that an individual’s stressors may have a short and/or long-term impact on the health of others. However, little is known about how HL influences the proliferation of stress within married couples. Drawing on nationally-representative data from 11 waves (1998-2018) of the Health and Retirement Study (n=9,000 individuals, 4,500 couples), we use age-based mixed models to examine how one’s own HL, spouse’s HL, or both spouses have HL shape the level and changes in depressive symptoms. For men, we find that their wives’ HL, their own HL, and both spouses having HL are each associated with an increase in depressive symptoms—and that the associations persist as spouses age. For women, we find that their own HL and both spouses having HL is associated with an increase in depressive symptoms. Furthermore, we find that the differences in women’s depressive symptoms between spouses who both have HL and those who do not have HL significantly declines with age. We also find no evidence to suggest that husbands’ HL is associated with wives’ depressive symptoms. Together, these findings suggest that the connections between spouses’ HL and their depressive symptoms are a dynamic process that unfolds differently by gender over time. Interventions that recognize the proliferation of stress associated with HL may help both individuals with HL and their spouses reduce their depressive symptoms.

